# The Effects of Aluminium Sulphate on Slag Paste Activated with Sodium Hydroxide and Sodium Silicate

**DOI:** 10.3390/ma13102286

**Published:** 2020-05-15

**Authors:** Taewan Kim, Sungnam Hong, Choonghyun Kang

**Affiliations:** 1 Department of Civil Engineering, Pusan National University, Busan 46241, Korea; ring2014@naver.com; 2 Department of Ocean Civil Engineering, Gyeongsang National University, Tongyeong 53064, Korea; snhong@gnu.ac.kr

**Keywords:** alkali-activated slag, aluminium sulphate, activators, alkaline environment

## Abstract

This study investigates the characteristics of alkali-activated slag cement using aluminium sulphate (ALS) as an activator. The alkalis NaOH and Na_2_SiO_3_ were used as additional activators (denoted by alkali) at 5% and 10% of the weight of the ground granulated blast furnace slag (GGBFS). Three types of activators were considered. The first was when ALS was used alone. For the second, ALS and 5% alkali were used together. The third was when ALS and 10% alkali were used. ALS was used at concentrations of 2%, 4%, 6%, 8%, and 10% based on binder weight. Experimental results show that when ALS was used as a sole activator, the activity of GGBFS was low and its strength was below 1 MPa. However, compressive strength was improved when 5% or 10% alkali and ALS were used at the same time. This was effective at improving mechanical and microstructural performance when used with an additional activator capable of forming a more alkaline environment than using ALS as a sole activator.

## 1. Introduction

Alkali-activated slag cement (AASC) activates ground granulated blast furnace slag (GGBFS) using several activators to promote hydration [1,2]. Studies on alkali-activated slag cement have been reported by various researchers until recently [3,4,5,6]. The nature of the activator is one of the major factors affecting the properties of AASC. The main activators used in previous studies were sodium carbonate (Na_2_CO_3_) [7], sodium hydroxide (NaOH) [8], and sodium silicate (Na_2_SiO_3_) [9,10,11]. These activators may be used alone or in combination [7,8,12,13,14]. The concentration of activator affects the initial behaviour (consistency and settling time) and strength of AASC. The alkaline activator of high pH value (>12) accelerates the hydration of GGBFS, shortening the setting time and increasing its strength [10]. Therefore, studies on the mechanical and microstructural properties of AASC according to the characteristics and use of various activators are continuing. In addition, various attempts are still being made to develop and apply new activators.

Compared to AASC, which used sodium hydroxide or sodium silicate as an activator, there were relatively few studies using sulphate-based activators. Additionally, there are not many sulphates used as activators at present. The most reported activators are sodium sulphate (Na_2_SO_4_) [15,16,17,18,19] and gypsum (CaSO_4_·2H_2_O) [20,21,22]. Often, studies of anhydride (CaSO_4_) [23] and hemihydrate (CaSO_4_·0.5H_2_O) [19] have also been reported. What has been confirmed through reports of previous studies to date is that the sulphate-based activator has a slow rate of hydration of AASC and low initial strength [24]. It is presumed that the cause of this is that the initial hydration of GGBFS according to the low pH value of the sulphate-based activator is very slow, and hence it takes a long time to activate. Recently, research results using magnesium sulphate as an activator were reported, but described very low mechanical properties [25]. Aluminium sulphate (ALS) has not been used as an activator. It is necessary to confirm whether ALS is applicable as an activator of AASC and how it affects mechanical and microstructural properties. ALS has long been used as a flocculants agent in water treatment. It has been used for a long time, and it is used in a variety of cheap and water treatment processes.

Discoveries of and applications for new activators are needed, along with investigations into their mechanical and microstructural properties. On the basis of the results of research into these different activators, we will clarify the characteristics of AASC and expand its use to various applications. In addition, the effect of mixing the various activators on the characteristics of AASC will be examined. The effect of this mixed activator is expected to help to obtain sufficient data to improve the performance of AASC.

The aim of the study was the experiment using ALS during the basic research to discover the new sulphate-based activator and its effectiveness. Two kinds of experiments were carried out for this purpose. First, we tested the properties of AASC using ALS as a sole activator. Second, the characteristics of AASC mixed with ALS were investigated using NaOH and Na_2_SiO_3_ as additional activators. Therefore, we investigated the effect of ALS as an activating agent of AASC and the effects of mixing ALS with conventional AASC.

## 2. Materials and Methods 

### 2.1. Materials

The composition of the GGBFS, as obtained through X-ray fluorescence (XRF) analysis, is summarised in Table 1. The fineness, density, and loss on ignition (LOI) values of the GGBFS used in the experiment were provided by the product supplier (KRT, Pohang, Korea). The ALS (Al_2_(SO_4_)_3_·14–18H_2_O) was obtained from SAMCHUN Chemicals. ALS is a white powder having a bulk density of 1.69 g/cm^3^ and a pH range of 3.0–4.0. Sodium hydroxide and sodium silicate were also obtained from SAMCHUN Chemicals. The sodium hydroxide was in the form of pellets with a purity of 98.0%, and sodium silicate was in liquid form with a silicate modulus Ms = 2.0. When sodium hydroxide and sodium silicate were used as activators, two concentrations were applied, 5% and 10% of the binder weight. The use of 5% alkali means 5% sodium hydroxide + 5% sodium silicate, and 10% alkali means 10% sodium hydroxide + 10% sodium silicate. In this experiment, no superplasticiser was used. Sodium hydroxide and sodium silicate were premixed in mixing water and left at room temperature for 6 h before use.

### 2.2. Experiments

The water–binder ratio of all AASC pastes was constant at 0.45. The weight of GGBFS used in one mixture was 3000 g. The following two factors were considered for the determination of the w/b ratio, and were determined through preliminary experiments. The first refers to the w/b ratio through investigations of previous studies [15,16,17,21,22,23]. The w/b range of alkali-activated cement using a sulphate-based activator was considered. Second, ALS was used as a coagulant in water treatment processes. Therefore, at a w/b ratio smaller than 0.45, it was difficult to prepare the sample because it was not mixed and molded. The experiments were divided into two types. The first used ALS as a sole activator. The second used ALS mixed with 5% or 10% of alkali. In both mixtures, ALS was used at concentrations of 2%, 4%, 6%, 8%, and 10% of binder weight.

The mixing time and order of blending followed ASTM C305 [26]. After compounding, the mixture was placed in a 25 × 25 × 25 mm^3^ mould [16] and stored in a chamber having a temperature of 23 °C ± 2 °C and a relative humidity of 90 ± 5%. After 24 h, the mould was removed, and samples were stored in chambers of the same temperature and humidity. The compressive strengths were measured according to ASTM C109 [27] at 1, 3, 7, and 28 d. The mean value from the three samples was used as the measurement value. In this experiment, a sample for compressive strength measurement of 25 × 25 × 25 mm^3^ was prepared to minimize drying shrinkage, cracking, and sample deformation due to the long setting time of paste and low initial compression strength. The setting time was measured according to the method of ASTM C191 [28]. In the case of sulfate, it was measured up to 7 d in consideration of the case where the setting time of AASC was not increased and hardened.

The pH measurement was performed on test bodies at 1, 3, and 28 d. Preparation of pH measurement samples was carried out according to the method of Rashad et al. [16], who performed AASC paste experiments using sodium sulphate.

For the measurement, the sample pieces were immersed in acetone for 12h and dried in a vacuum desiccator for 24 h. The X-ray diffractometer (XRD) used Xpert3 from PANalytical (Malvern, UK). The measurement range was 5° to 60° (2θ), the step size was 0.017° (2θ), and the operating environment was 40 kV, 40 mA. Mercury intrusion porosimetry (MIP) was analysed by Micromeritics’ AutoPore IV 9500 (GA, USA) to analyse the pore structure and size of the sample. The measurement conditions were a contact angle of 130 °, a mercury density of 13.534 g/ml, and a surface tension of 485 dyn/cm. For thermal analysis, DSC 800 (MA, USA) from Perkin Elmer was used to measure thermogravimetry (TG)/Derivative thermogravimetry (DTG). The analysis was conducted in an N_2_ gas environment; the measurement temperature range was 30 °C to 800 °C; and the heating rate was 20 °C/min. Scanning electron microscopy/backscattered electron (SEM/BSE) for observation of hydration reactants was used by Zeiss SUPRA 40 (Oberkochen, Germany). The observations were performed in the high vacuum mode with an accelerating voltage of 15 kV. The atomic ratio was measured by performing energy-dispersive X-ray spectrometry (EDS) analysis on the hydration reactant.

## 3. Results and Discussion

### 3.1. Setting Time

**Figure 1:**
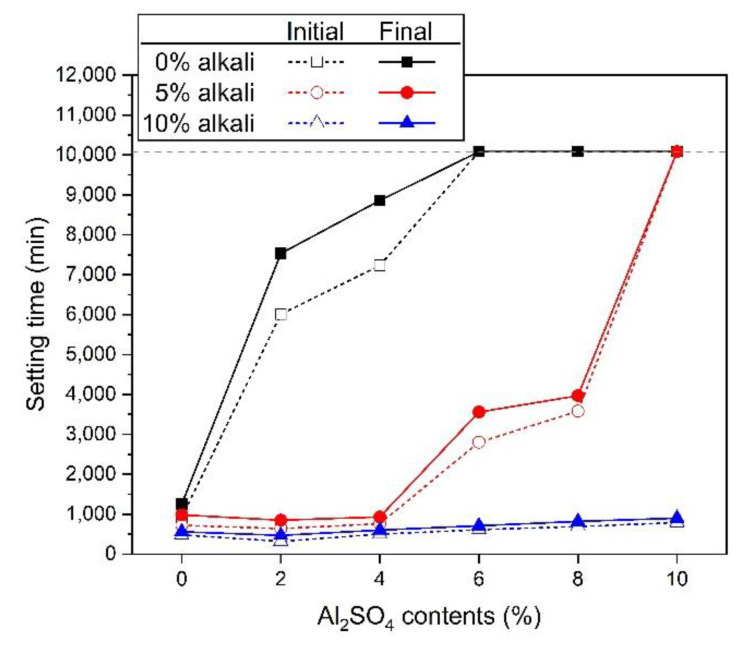
Setting time of each mixture.

Figure 1 shows the measured value of setting time for the activator. For 0% alkali samples using ALS as the sole activator, the final setting time of 0% ALS was 1250 min. However, from 2% ALS, the initial and final setting times increase rapidly. Additionally, 6%–10% ALS was not setting until 7 days in. It can be seen that the use of ALS alone has little effect on the activation of GGBFS particles and thus has little effect on the formation of hydration reactants. As a result, the setting is delayed and this phenomenon becomes clear as the concentration of ALS increases. In 5% alkali samples, 2% ALS had the shortest initial and final setting times. Then, as the amount of ALS increased from 4% to 8%, the setting time increased rapidly. However, 10% ALS was not setting until 7 d. All 10% alkali samples were setting before 1 d. 2% ALS showed the fastest setting time. The setting time gradually increased as the concentration increased to 4%–10% ALS.

ALS increases the setting time. However, when used with 5% and 10% alkali, the setting time decreases. The setting time increases as the amount of ALS increases, despite the use of 5% and 10% alkali. Therefore, the increase in the amount of ALS prevents the formation of hydration reactants of the GGBFS particles [24]. The tendency to increase the setting time of ALS is consistent with the results of alkali-activated slag cement using sulphate-based activators reported by previous studies [20].

### 3.2. Compressive Strength

**Figure 2:**
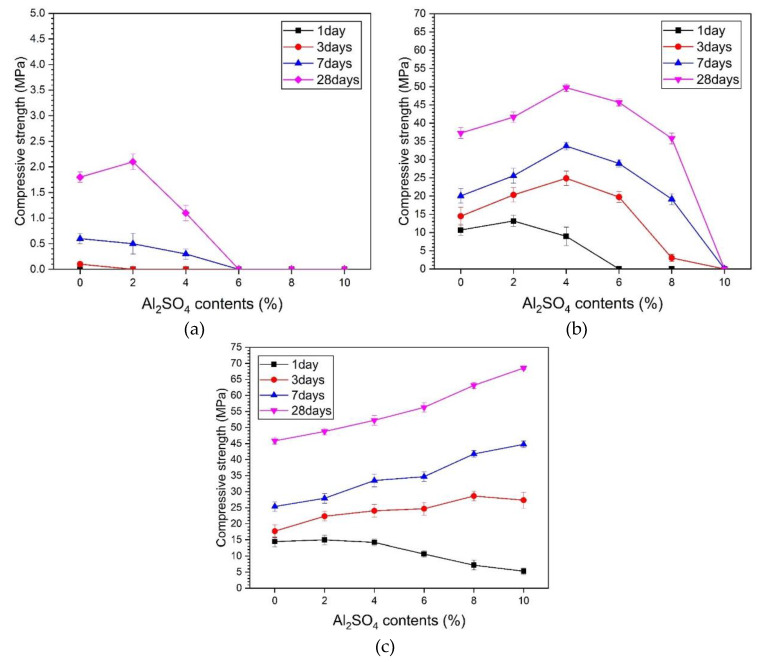
Compressive strength: (**a**) ALS (without alkali), (**b**) 5% alkali with ALS, and (**c**) 10% alkali with ALS.

Figure 2 shows the compressive strength results measured at different ages for different activators. Figure 2a shows the results of samples using ALS as a single activator (0% alkali). The strength did not improve, even though the ALS concentration increased. In the sample without ALS, at day one, strength was unmeasurable, and the strengths of days 3, 7, and 28 were 0.1, 0.2, and 0.9 MPa. In the 2% ALS sample, the values of day one and day three were unmeasurable, and strength for day 7 and day 28 was 0.3 and 1.1 MPa respectively. The specimens with more than 4% ALS were 0 MPa in strength at all ages. Both the initial strength and late strength of AASC using ALS as a sole activator were either very low or not measurable. The samples with ALS showed a lower compressive strength tendency than the sulphate-based activators used in previous studies did [23,24,29,30]. The use of ALS as a sole activator has a low activating effect of GGBFS, consequently delaying setting and slowing hardening. Therefore, it is necessary to consider mixing ALS with additional activators.

Figure 2b shows the compressive strengths of the samples using an activator mixed with 5% alkali and ALS. In 5% alkali, the relationship between strength and ALS concentration shows various characteristics at each age. The 1-day compressive strength was maximal for 2% ALS. The strength gradually decreased from 2% ALS to 6% ALS. The strength value at day one of 6%–10% ALS was unmeasurable. However, at days 3, 7, and 28, it showed its highest values at 4% ALS concentration. After 4% ALS, the strength gradually decreased and finally could not be measured by un-hardening at 10% ALS. As the alkali concentration of the mixture increases, the GGBFS-activating action of ALS is promoted, which affects the improvement of compressive strength. In addition, as shown in Figure 2b, there was a specific ALS concentration at which the maximum strength occurred in the activator mixed with 5% alkali and ALS simultaneously. Compared with that in Figure 2a, where ALS was used as a sole activator, the improvement in strength was confirmed in Figure 2b, where 5% alkali and ALS were used. However, as the concentration of ALS increases, the strength improvement effect decreases.

Figure 2c shows the results from ALS mixed with 10% activator. The day one strength showed a slight decrease after 2% ALS but a value higher than 5 MPa. The compressive strength increased between day 3 and day 28 with increasing ALS concentration, and the maximum value was measured at 10% ALS. The 28-day strength of 10% alkali and 0% ALS was 45.8 MPa, and 10% ALS was increased to 68.5 MPa.

From the results in Figure 2, it was found that using 5% and 10% alkali and ALS together was more effective for strength improvement than using ALS as the sole activator. It is presumed that the effect of ALS on the hydration of GGBFS and the formation of reaction products in a highly alkaline environment are enhanced.

Table 2 shows the relative change in the strength value at each ALS concentration when compared to the without-ALS sample for the strength values of 5% and 10% alkali. The ALS concentrations at 5% alkali showing the highest intensity increase at each age are as follows. At day one, it was 2% ALS, and for days 3, 7, and 28—4% ALS. With 10% alkali, the highest intensity growth rate at each age was 2% ALS at 1 d, 8% ALS at 3 d, and 10% ALS at 7 and 28 d. As the concentration of activator increased from 5% to 10%, the concentration of ALS, which had the effect of increasing the strength, also increased. Thus, an alkaline environment affects the GGBFS hydration-promoting action of ALS. When the concentration of the activating agent increases from 5% to 10%, a highly alkaline environment is created, which enables hydration of GGBFS at a higher concentration of ALS. The use of 5% and 10% activators accelerates the activation of GGBFS, thereby eluting various ions and improving the hydration reaction [31,32,33,34].

The compressive strength tendencies of 5% and 10% alkali and ALS mixed samples are consistent with previous studies in which the initial strength is improved by mixing Na_2_SO_4_ with lime and alkaline PC clinker [24]. In a previous study in which sodium silicate-based AASC paste was mixed with up to 6% gypsum, the intensity increased as the amount of gypsum increased [20]. On the basis of the results of previous studies and the present study, sulphate activators have the effect of improving strength when used together in an alkaline environment rather than alone. Therefore, ALS can be used as an activator of AASC in a highly alkaline environment. This means that concurrent use of ALS with highly alkaline activators is more effective than with ALS alone.

### 3.3. Hydration Reactants Products

**Figure 3:**
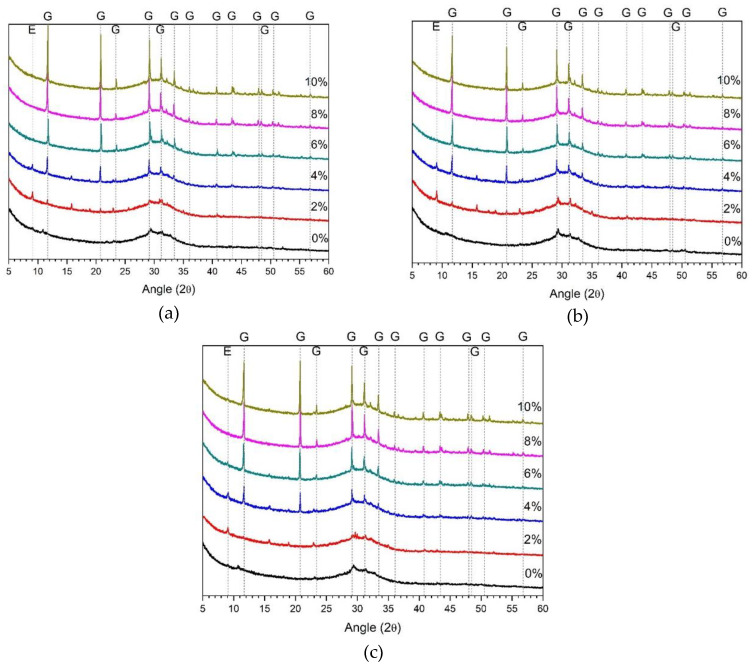
XRD analysis of ALS-only samples (without alkali) at (**a**) day 1, (**b**) day 3, and (**c**) day 28. E: ettringite; G: gypsum.

Figure 3 shows the 1, 3, and 28 d XRD results of samples using ALS as the sole activator. Ettringite was observed in the 2% and 4% samples, but was not observed in the ALS concentrations above 6%. The ettringite showed the highest peak in the 2% ALS. Ettringite has also been reported as the major hydration product in the study of AASC using sodium sulfate as the activator [22,23]. Ettringite was also observed in this study using ALS as an activator.

However, 2% ALS shows a higher ettringite peak than does 4% ALS. The highest compressive strength in Figure 1 was measured at 2% AS. Therefore, it can be concluded that ettringite affects the mechanical properties.

The gypsum peak rapidly increases from 4% ALS to 10% ALS. This tendency was observed at 1, 3, and 28 d. Therefore, it is considered that the amount of gypsum is increased as the amount of ALS used as an activator becomes excessive. The compressive strength of the 4%–10% ALS with a sharp increase in the gypsum peak was easily broken and could not be measured. Therefore, the presence of excessive gypsum is presumed to suppress the development of strength. This is consistent with the results of previous studies, suggesting excessive gypsum is the cause of strength reduction [22,23].

**Figure 4:**
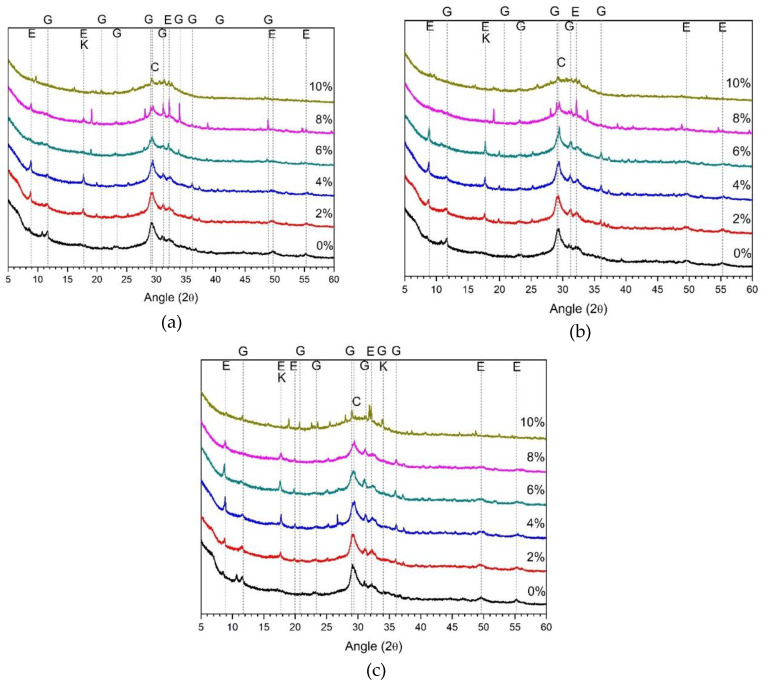
XRD analysis of 5% alkali with ALS at (**a**) day 1, (**b**) day 3, and (**c**) day 28. E: ettringite; G: gypsum; C: C–S–H gel and calcite; K: katoite.

Figure 4 shows the XRD results of 5% alkali and ALS mixed samples. The major reaction products of 5% alkali and ALS mixed samples are ettringite, calcium silicate hydrate (C–S–H) gel, and gypsum. Previous studies using sodium sulphate or calcium sulphate as activators have also referred to ettringite and C–S–H as the main reactants [15,16,17,18,19]. Calcium silicate hydrate is a new hydration product that was not observed in samples using ALS as the sole activator. The 5% alkali promotes the hydration of GGBFS and increases the elution of calcium, aluminium, and silica. It also reacts with gypsum derived from ALS to form ettringite [19].

The ettringite and gypsum peaks shown in Figure 4 were reduced when compared to the XRD results using ALS alone shown in Figure 3. In contrast, C–S–H increased. The trend of these reactants was observed after 1, 3, and 28 d, as shown in Figure 4. In Figure 2b, the maximum strength of the sample mixed with 5% alkali and ALS was confirmed at 4% ALS. The highest ettringite peak in Figure 4 is seen at 4% ALS. The C–S–H peak also increases with increasing ALS content, and the highest peak is observed at 4% ALS. Thereafter, the C–S–H peak gradually decreases until 10% ALS. In Figure 4, after 1, 3, and 28 d, 10% ALS showed low ettringite and gypsum peaks because GGBFS activation was rarely achieved.

**Figure 5:**
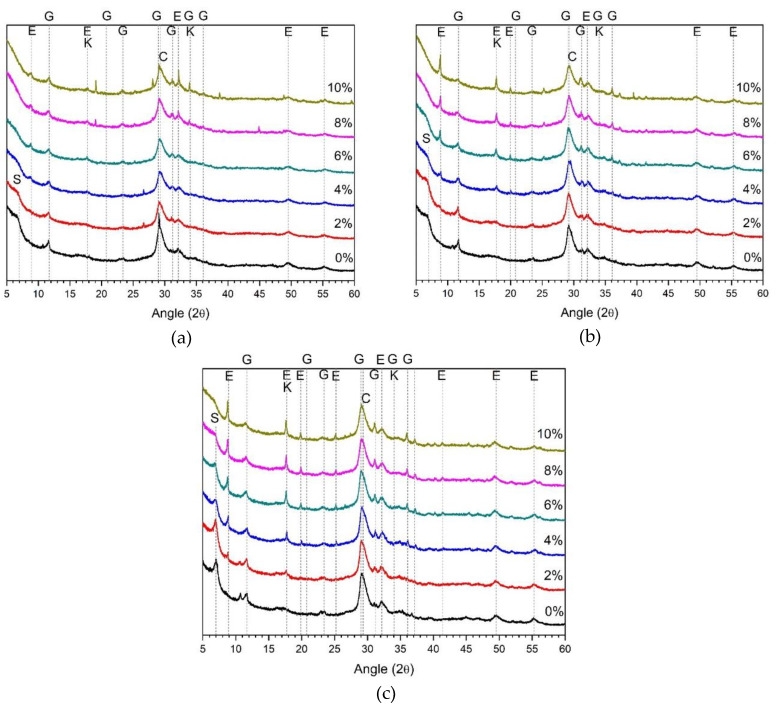
XRD analysis of 10% alkali with ALS after (**a**) 1 d, (**b**) 3 d, and (**c**) 28 d. E: ettringite; G: gypsum; C: C–S–H gel and calcite; S: strätlingite; K: katoite.

Figure 5 shows the XRD results of a sample mixed with 10% alkali and ALS. The ettringite, gypsum, and C–S–H gels observed in Figure 4 are also observed therein. As shown in Figure 2c, the highest compressive strength was observed at 10% ALS. In Figure 5, the highest ettringite peaks and the lowest gypsum peaks were observed at 10% ALS after 1, 3, and 28 d. In addition, C–S–H peaks higher than those of Figure 4 were observed.

Strätlingite was observed, as shown in Figure 4c. Strätlingite and katoite are often observed in AASCs with the addition of Al-rich salts or additional aluminium ions [35,36,37]. As the concentration of ALS increases, the strätlingite peak gradually decreases. This is a phenomenon not observed in Figure 4. This is due to the increased aluminium elution of GGBFS and ALS at high alkali concentrations. Unstable strätlingite is formed in a high-pH environment [38]. In general, strätlingite is also found with C–S–H, hydrogarnet, and katoite [39]. However, the inclusion of ALS reduces the pH and reduces the production of strätlingite.

The XRD results for the reaction products in Figure 3, Figure 4 and Figure 5 show that the highly alkaline environment facilitates the separation of aluminium and sulphate from the ALS. The result is the generation of strätlingite and gypsum. Hydration of GGBFS is also promoted in a highly alkaline environment. The calcium, aluminium, and silica are eluted from GGBFS. When ALS is used as the sole activator, these eluting ions react with gypsum to produce ettringite. However, in highly alkaline environments, the formation of C–S–H gels is greater than that of ettringite. As shown in Figure 3, when ALS was used as a sole activator, ettringite and gypsum peaks were the most common, and C–S–H gel was not observed. Ettringite, C–S–H gel, and gypsum are shown together in Figure 4, where 5% alkali and ALS are mixed. As shown in Figure 5, ettringite and gypsum were rarely observed, and C–S–H gel was observed. Therefore, hydration reaction products can be assumed to be influenced by a highly alkaline environment and the ALS concentration.

Despite the high ALS doses used in this study, unreacted ALS in the hydration products was not identified as a crystalline product in these samples. This is because most of the ALS reacts to form ettringite or gypsum. Aluminium eluted from ALS and GGBFS forms strätlingite [35,36,37]. It is also assumed that some aluminium was adsorbed onto C–S–H gel and consumed to form C–A–S–H gel [35,38]. Unreacted aluminium may also be present as gibbsite. However, gibbsite and C–A–S–H gels are not shown in Figure 3, Figure 4 and Figure 5 because they are amorphous materials not observable by XRD.

In the XRD results, the use of ALS alone with no alkali produced some ettringite and no C–S–H gel was observed. ALS first mixes with calcium eluted from GGBFS to form gypsum. Therefore, calcium is primarily consumed to form gypsum, which is insufficient to form C–S–H gel or ettringite, which is a general hydration of AASC. However, simultaneous use of 5% and 10% alkali with ALS dramatically increases the activation of GGBFS. Thus, calcium elution increases from GGBFS. Increasing calcium gradually reduces gypsum formation and increases ettringite and C–S–H gel formation. This reaction is evident as the concentration of alkali increases from 5% to 10%. As a result, the high alkaline environment promotes the activation reaction of GGBFS and rapidly increases the elution of ions such as calcium, thereby changing the type of hydration reactants.

### 3.4. Pore Structure

**Figure 6:**
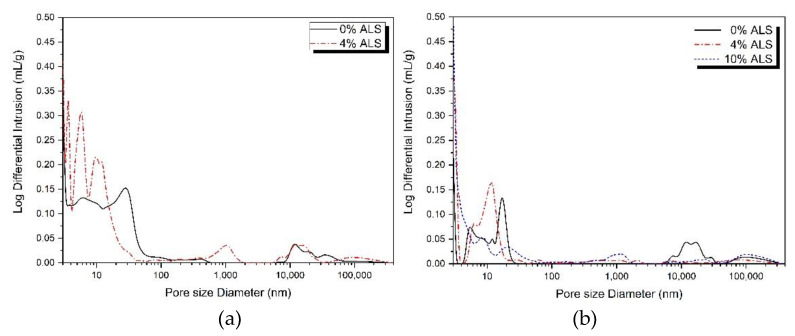
Pore structure analysis: (**a**) 5% alkali + ALS and (**b**) 10% alkali + ALS.

Figure 6 shows the results of MIP measurements to confirm the pore structures of samples mixed with ALS alone, with 5% alkali, and with 10% alkali. Samples using ALS as a sole activator could not be assessed using MIP because of their low compressive strengths. MIP measurements were made using a mixture of 0% or 4% ALS with 5% alkali. 10% ALS mixed with 5% alkali was excluded from MIP measurements because it was not hardened due to unsetting. In 10% alkali and ALS mixed samples, MIP analysis was performed on no-ALS, 4% ALS, and 10% ALS samples.

It is generally reported that as the concentration of the activator in AASC increases, more reaction products are produced and a dense matrix with reduced pore volume and diameter is formed [11]. In Figure 6a, it can be seen that there are more micropores of less than 100 nm in 4% ALS compared with those in 0% ALS. Additionally, in Figure 6b, it can be seen that the size and number of pores gradually increase in the order of 10% ALS < 4% ALS < 0% ALS.

Table 3 shows the analysis of pore sizes below 1000 nm [40], and the total porosity is shown in the graphs of Figure 6.

In Table 3, it can be seen that total porosity decreased slightly from 30.86% to 29.12% at 0% ALS and 4% ALS, respectively, in 5% alkali samples. The 10% alkali samples had 15.71%, 16.43%, and 15.94% total porosity at 0%, 4%, and 10% ALS, respectively. The total porosity of 10% alkali samples was reduced to about half the total porosity of 5% alkali samples. However, the change in total porosity due to the inclusion of ALS was not significant. Therefore, it is estimated that the effect on the total porosity of including ALS is small. Table 3 clearly shows the effect of ALS on the distribution of pore size.

When the amount of ALS is increased from 0% to 4% in the mixture with 5% alkali, large capillary pores increase slightly, medium capillary pores decrease, and gel pores increase. A similar tendency was observed in the sample mixed with 10% alkali and ALS. That is, when the concentration of ALS was increased to 10%, large capillary pores increased slightly, medium capillary pores decreased significantly, and gel pores increased steeply. The inclusion of ALS causes the decrease of medium capillary pores and the increase of gel pores. In particular, the increase in the number of gel pores means the generation of dense hydration reactants, such as C–S–H gel [40]. Therefore, ALS helps to activate GGBFS in a highly alkaline environment and reacts with ions eluted from GGBFS to form dense hydration reactants. This results in an increase in the compressive strength and a change in the pore structure.

Activated slag studies using gypsum and CaO [22] and slag studies using lime and gypsum [21] are consistent with reported reductions in the number and size of pores. In this study, the mixing of ALS was found to affect the decrease of pore size. However, despite the decrease of medium capillary pores and the increase of gel pores, large capillary pores and the total number of pores were found to be slightly increased. This can be attributed to the excessive expansion of gypsum [22]. Furthermore, ALS can be interpreted as having a greater effect on the size reduction of pores than on their overall number. This is because, as mentioned above, the reactivity of the ALS is increased to form dense hydration reactants.

### 3.5. Thermal Analysis

**Figure 7:**
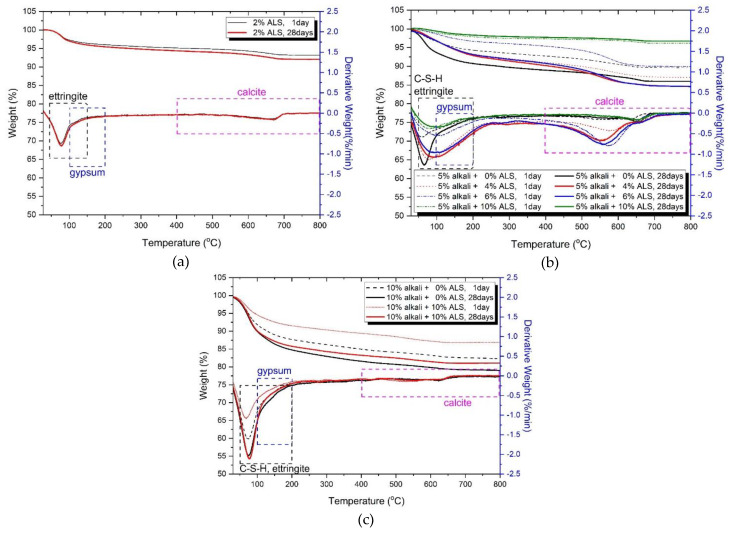
TG and DTG analysis for (**a**) 2% ALS (without alkali), (**b**) 5% alkali + ALS, and (**c**) 10% alkali + ALS.

Figure 7 shows the thermal analysis (TG/DTG) results for samples after 1 and 28 days. The reactants observed in Figure 7 are summarised in Table 4.

Figure 7a shows the TG/DTG results for 2% ALS with the highest intensity among samples using ALS as the sole activator. The change in the weight loss rate of ettringite and gypsum at 1 and 28 days is insignificant. Therefore, even if the age increases, the hydration reaction of GGBFS is hardly achieved. This is because the ALS is less effective at activating GGBFS.

Figure 7b shows the TG/DTG results for 0%, 4%, 6%, and 10% ALS in 5% alkali samples. Activation of GGBFS is promoted in a highly alkaline environment including 5% activator, resulting in a difference in the amount of hydration reaction product produced by ALS. In Figure 7b, the 5% alkaline + 0% ALS increased the ettringite weight loss rate at 28 days compared with that at 1 day. This is due to the continuous hydration of GGBFS with increasing age. The 5% alkali + 4% ALS with the highest intensity measured showed a small difference in the rate of weight reduction for C–S–H/ettringite between days 1 and 28. However, the 5% alkali + 6% ALS increases the difference in the weight reduction rate between days 1 and 28 for C–S–H/ettringite, and 5% alkali + 10% ALS again has a smaller weight reduction rate. In particular, 10% ALS showed the lowest weight loss rate and very little C–S–H/ettringite production. These results support low ettringite and C–S–H peak results at 10% ALS, as shown in Figure 7b.

Figure 7c shows the TG/DTG results for 10% alkali and ALS. The weight reduction rate of C–S–H/ettringite with 10% ALS was higher than that in 0% ALS. This means that 10% ALS forms more hydration reaction material. The high ettringite and C–S–H peaks shown in the XRD results in Figure 5 are consistent with those observed. In the TG/DTG results, 5% and 10% alkali are more effective for hydration reactions than is ALS in the initial (1 day) hydration reaction. However, as the age increases (to 28 days), the influence of ALS becomes clear. This tendency can be confirmed by the fact that the rate of increase in strength on day 28 is larger than that on day one, as shown in Table 2. Therefore, ALS has a great influence on the formation of hydration reaction products as age increases in a high-alkali-concentration environment.

### 3.6. Values of pH

**Figure 8:**
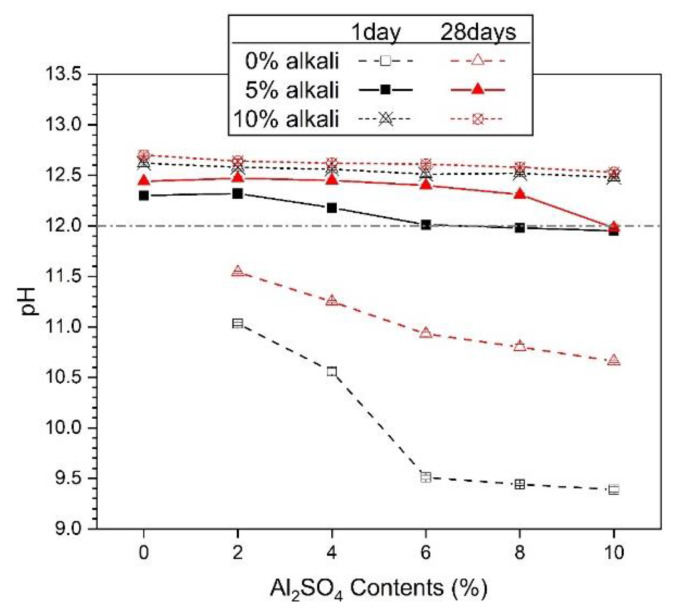
The pH values for AASC pastes with activator.

Figure 8 shows the pH values measured at days 1 and 28. In Figure 8, when ALS was used as the sole activator, the pH values in the day 1 and day 28 samples ranged from 9.39 to 11.54. As the amount of ALS increases, the pH decreases. At day one, more than 6% ALS will cause a sharp decrease in pH value. However, after 28 days, the pH value was higher than that after 1 day. In particular, 6%–10% ALS samples, which showed low pH values after 1 day, showed very high pH increase rates after 28 days.

Samples of ALS mixed with 5% alkali showed pH values in the range of 11.96–12.47. The pH value increased for all ALS concentrations as time increased from day 1 to 28. However, after showing the highest pH value at 2% ALS, it gradually decreases as the concentration of ALS increases. Samples mixed with ALS with 10% alkali were measured in the range of 12.48–12.73. The decrease in pH as the concentration of ALS increases tends to be gentle. Mixing the alkali and increasing the concentration decreases the variability of the pH value according to the amount of ALS with the improvement of the pH value.

The pH of the activator in AASC is one of the important influence factors in the hydration process of GGBFS. Previous studies have reported that a pH higher than 11.5 is required to facilitate hydration of GGBFS, and that hydration rarely progresses at values lower than 9.5 [18,46]. It is also known that the high-pH environment created by a high concentration of activator further promotes the hydration of GGBFS [18,32,33,34]. Increasing the hydration reaction of GGBFS promotes the formation of hydration reaction products, thereby densifying the matrix and improving mechanical strength [32]. However, when the pH value is less than 12, it has been reported that the hydration of GGBFS is delayed, and this results in low mechanical strength [32].

In Figure 8, samples using ALS as the sole activator showed pH values less than 12 for all ALS percentages. The pH values at 28 days were still below 12. As a result, as shown in Figure 2a, the compressive strength was almost unmeasurable. In Figure 8, the pH values at day one of less than or near 12 were from samples between 6% and 10% ALS. In Figure 2b, the daily strength of 6%–10% ALS was 0. In 10% ALS, the pH value was less than that after 12 at 28 days, and the compressive strength values after 28 days in Figure 2b were 0.

However, all samples with 10% alkali + ALS had pH values greater than 12. Therefore, as mentioned in previous studies, when the pH value is less than 12, the hydration of GGBFS is hardly achieved, and the strength is not measurable. In addition, when the pH value is close to 12, the compressive strength is small. However, comparing Figure 8 and Figure 2, the rate of increase in pH and the rate of increase in strength were not proportional. For example, on day 28, Figure 8 shows a slow decrease in the pH value from 0% to 10% ALS, and the compressive strength shown in Figure 2 decreases with a similar slope. However, the compressive strength at day 28 increases in contrast to the decrease in pH value. Thus, it is clear that pH is one of the factors affecting the hydration and strength of GGBFS, but the relationship is not directly proportional. It can be assumed that there are other characteristics that affect the strength improvement, such as the nature of GGBFS, curing conditions, hydration products and pore structures.

The pH measurement results in Figure 8 show that 5% and 10% alkali increase the pH, and ALS decreases the pH. Therefore, a combination of alkali and ALS can create a pH environment in excess of 12 that can promote hydration of GGBFS. As a result, ALS exhibits a synergistic effect that further promotes hydration of GGBFS in highly alkaline environments, improving its mechanical properties.

### 3.7. Microstructure (SEM)

**Figure 9:**
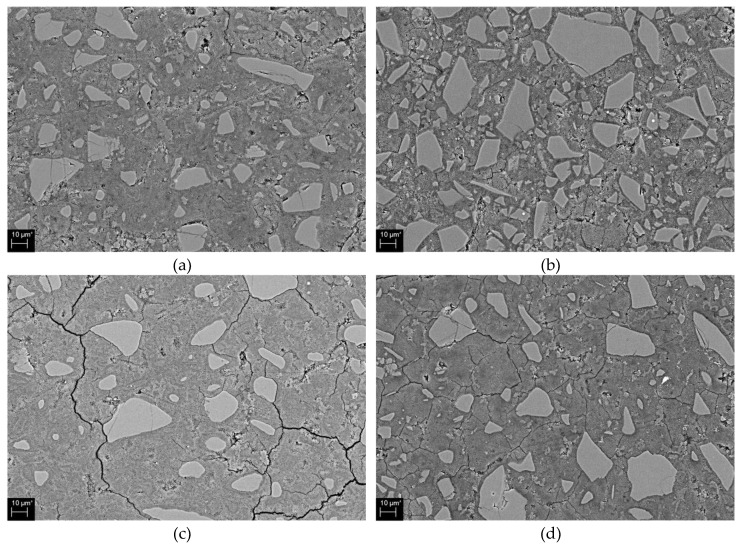
SEM/BSE image showing (**a**) 5% alkali + 0% ALS, (**b**) 5% alkali + 4% ALS, (**c**) 10% alkali + 0% ALS, and (**d**) 10% alkali + 10% ALS.

Figure 9 shows scanning electron microscopy/backscattered electron (SEM/BSE) images for 5% alkali with 0% and 4% ALS (Figure 9a,b, respectively), and 10% alkali with 0% and 10% ALS (Figure 9c,d, respectively). The cracks observed in the image in Figure 9 occurred during the hydration, drying, and polishing of the sample. Comparing 4% and 0% ALS at 5% alkali, the former shows a denser matrix with fewer voids. Ten percent alkali is similar to 5% alkali. That is, 10% ALS shows a more compact matrix than does 0% ALS. The 10% alkali sample shows a denser matrix than does the 5% alkali sample. As the concentration of activator increases, the hydration of GGBFS is promoted to form a dense matrix, which has already been mentioned in previous AASC studies [18].

Table 5 shows the results of EDS analysis on Ca/Si and Al/Si. To obtain the ratio of Ca, Al, and Si by EDS analysis, three points were arbitrarily selected from hydration reactants shown in the SEM images of Figure 9.

In 5% alkali samples, 0% ALS shows lower Al/Si and higher Ca/Si than does 4% ALS. At 10% alkali, 10% ALS shows higher Al/Si and lower Ca/Si than does 0% ALS. The presence of ALS increases Al/Si and decreases Ca/Si in the hydration reactants. This is because the aluminium ions added by the ALS increase the Al concentration in the hydration reactants. Thus, this increases the absorption of the aluminium ion in C–S–H gel [38,47]. Furthermore, the presence of Ca/Si decreases as the concentration of alkali increases from 5% to 10%. As the proportion of ALS is increased, Ca/Si tends to decrease. This is due to the increased silica concentration in the activator [48,49].

## 4. Conclusions

Experimental results of the mechanical and microstructural characteristics of alkali-activated slag paste using ALS as an activator are summarised as follows.

Activation of GGBFS was very low when ALS was used as the sole active agent. Therefore, the compressive strength was barely measurable. However, when ALS is used in combination with 5% or 10% alkali, the strength is improved. This means that ALS has the effect of promoting hydration of GGBFS in a highly alkaline environment. In particular, the maximum compressive strength was obtained at 4% ALS for 5% alkali and 10% ALS for 10% alkali. Therefore, the higher the alkalinity of the mixed water, the more the GGBFS-activating action of ALS is promoted.

When ALS was used as a sole activator, gypsum was the major hydration reactant, and ettringite was found in some samples. When both alkali and ALS are used, ettringite, C–S–H gel, and gypsum are observed. In particular, using mixed alkali and ALS activators rapidly reduces gypsum and increases ettringite. These changes in hydration reactants become clearer as the concentration of alkali increases from 5% to 10%. Therefore, the mixed use of ALS and alkali promotes the formation of C–S–H gel and ettringite, thereby reducing the pore diameter and total porosity, consequently affecting the formation of a dense matrix. The tendency to decrease the pore diameter and total porosity can be confirmed through MIP analysis, which means that the strength of the samples is increased by densification of the matrix. As a result, medium capillary pores (50–10 nm) decrease, and gel pores (<10 nm) increase. The dense hydration reactants were identified by TG/DTG and SEM/BSE. The inclusion of ALS in particular increased the presence of Al/Si in the hydration reactants.

ALS could not be used as a sole activator because of its having a lower effect than calcium sulphate or sodium sulphate. However, when used in conjunction with an additional alkali to form a highly alkaline environment, ALS improves the mechanical properties of alkali-activated slag paste.

The ALS reacts immediately with calcium eluted from GGBFS and is mostly consumed to form gypsum. As a result, the lack of calcium causes delayed ettringite or C–S–H gel formation. However, the use of 5% and 10% alkali promotes the activation of GGBFS, which leads to a sharp increase in calcium elution. Eluted calcium is sufficient to react with ALS to form gypsum and to form ettringite and C–(A)–S–H gels. However, an increase in ALS again causes calcium deficiency. As a result, alkali promotes the activation of GGBFS and increases the elution of calcium, thereby affecting the formation of ALS and hydration reactants.

## Figures and Tables

**Table 1:** Properties of GGBFS.

Raw Material	Chemical Components (%)							Density (g/cm^3^)	Fineness (m^2^/kg)	LOI (%)
	SiO_2_	Al_2_O	Fe_2_O	MgO	CaO	K_2_O	SO_3_			
GGBFS	37.21	9.07	0.54	3.69	43.73	0.71	3.52	2.81	420	0.73

**Table 2:** Relative strength change rate compared with the no-ALS sample.

Level	Curing Ages (day)							
ALS Contents (%)	5% Alkali				10% Alkali			
	1	3	7	28	1	3	7	28
2	123.0	140.3	127.5	111.7	103.7	126.2	110.0	106.3
4	83.7	171.7	168.2	133.4	98.2	135.8	131.9	113.8
6	-	136.2	144.3	122.6	73.6	139.4	136.5	122.7
8	-	20.9	95.6	96.0	49.5	161.7	164.5	137.6
10	-	-	-	-	36.5	154.5	176.3	149.4

**Table 3:** Pore size analysis.

Level	5% Alkali		10% Alkali		
ALS	0%	4%	0%	4%	10%
Total porosity (%)	30.86	29.12	15.71	16.43	15.94
Large capillary pores (10000–50 nm), (%)	4.42	6.61	3.47	6.35	8.09
Medium capillary pores (50–10 nm), (%)	39.87	24.20	39.02	29.85	11.74
gel pores (<10 nm), (%)	55.71	69.19	57.52	63.80	80.17

**Table 4:** Temperature ranges for identifying the phases via TG (or DTG), DTA and DSC.

Phase	Temperature Ranges of Weight/Endothermic/Exothermic Change
C–S–H	50–200 °C [41] 120–145 °C [42] 106 ± 4 °C due to first loss of water [43] 29 ± 4 °C for due to second loss of water [43]
Ettringite	110–150 °C [44] 80–130 °C [43]
Gypsum	100–200 °C [22] 80–220 °C (max. 167 °C) [22]
Calcium carbonate (calcite)	720–760 °C [42] 400–800 °C [45]

**Table 5:** Component ratios of hydration reactants.

Atomic Ratios	5% Alkali (Without ALS)	5% Alkali + 4% ALS	10% Alkali (Without ALS)	10% Alkali + 10% ALS
Al/Si	0.34	0.40	0.38	0.41
Ca/Si	1.08	0.92	1.02	0.93
